# Atrioventricular Block in Pregnancy: 15.8 Seconds of Asystole

**DOI:** 10.7759/cureus.10720

**Published:** 2020-09-29

**Authors:** Taylor Sullivan, Anna Rogalska, Leticia Vargas

**Affiliations:** 1 Obstetrics and Gynecology, University of the Incarnate Word School of Osteopathic Medicine, San Antonio, USA; 2 Obstetrics and Gynecology, Metropolitan Hospital, San Antonio, USA

**Keywords:** pregnancy and heart disease, atrio-ventricular block, permanent pacemaker implantation (ppm), high degree av block, second degree heart block

## Abstract

Atrioventricular (AV) block in pregnancy is infrequently encountered and there is little management guidance available. We present a case of a 24-year-old G3P1011 at 24 weeks' gestation who presented to the obstetrics and gynecology clinic complaining of palpitations, fatigue, and dyspnea on exertion. Cardiology workup including an electrocardiogram (ECG) and Holter monitor detected second-degree type II (Mobitz) AV block with the longest asystole event lasting 15.8 seconds.

A St. Jude's dual-chamber pacemaker (Abbott Laboratories, Abbott Park, IL) was implanted immediately. Standard radiation precautions were taken with additional shielding for the fetus. The patient experienced significant improvement in her symptoms. The patient went into labor at 37 3/7 weeks. Due to non-reassuring fetal heart tones, a cesarean section was performed, and a healthy baby girl was born.

The management of heart block in pregnancy can be divided into involving those who are symptomatic and those who are asymptomatic. Symptoms of heart block can include palpitations, fatigue, dyspnea, and/or syncope; the presence of these symptoms warrants the placement of a pacemaker, preferably during pre-pregnancy or during the first two trimesters, as high-grade heart block is associated with significant mortality. Those who are in their last trimester or postpartum should consider the use of a temporary pacemaker as heart block could be due to pregnancy-related cardiovascular changes.

For women with heart block, labor and delivery could result in worsening of bradycardia due to uterine contractions displacing blood into the central circulation. Most women with heart block do well in labor and delivery and having a pacemaker is not necessarily an indication for a cesarean section.

## Introduction

Atrioventricular (AV) block in pregnancy is infrequently encountered. In this report, we present a case of a 24-year-old G3P1011 who presented with palpitations. She was diagnosed with second-degree type II (Mobitz) AV block, high-grade, and immediately had a dual-chamber pacemaker implanted. There are no established guidelines on the management of pregnant women with AV block, nor a specified procedure for pacemaker implantation for patients with a gravid uterus. Here, we describe our case and provide a literature review relating to the background, diagnosis, and management of heart block in pregnancy.

## Case presentation

A 24-year-old G3P1011 presented for a routine prenatal checkup at 24 weeks' gestation. She denied uterine cramping, vaginal bleeding, and confirmed fetal movement. Fetal heart rate, the estimated gestational weight, and fundal height were within normal limits. However, she complained of severe fatigue and “skipping and pausing” of the heart. The patient admitted that these sensations had occurred a few times in the past, but that they had never lasted more than five minutes. They were now occurring almost daily and for longer periods of time. The pertinent first-trimester screening results included blood type A Rhesus negative and rapid plasma reagin (RPR) positive; she had a history of treated syphilis prior to the pregnancy and had received no further treatment.

She began experiencing shortness of breath, dyspnea on exertion, and mild chest pain and was subsequently referred to see a cardiologist. Her only medications were prenatal vitamins and her review of systems was otherwise negative. Her blood pressure was 118/60 mmHg, heart rate was 109 beats per minute, and SpO_2_ was 98% on room air. Her exam was within normal limits.

**Figure 1:**
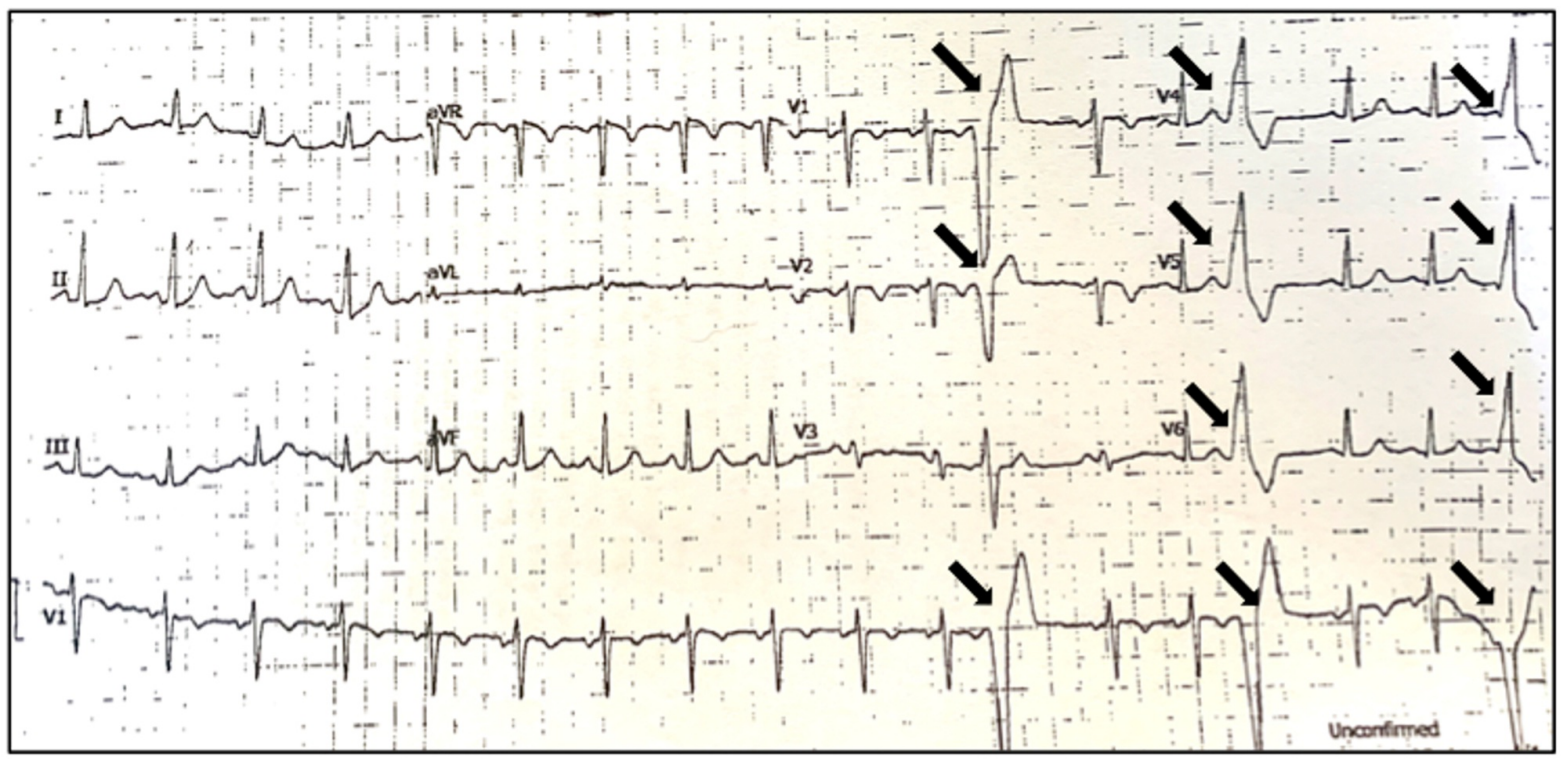
In-office ECG showing premature ventricular complexes ECG: electrocardiogram

An electrocardiogram (ECG) was performed (Figure 1), which revealed sinus tachycardia with frequent, isolated premature ventricular complexes (PVCs).

A transthoracic echocardiogram (TTE) was performed, which showed a left ventricular ejection fraction of 66% (normal range: 55-70%) [1] and mitral valve prolapse. A 14-day Holter monitor was ordered to evaluate the frequency of abnormal rhythms.

**Figure 2:**
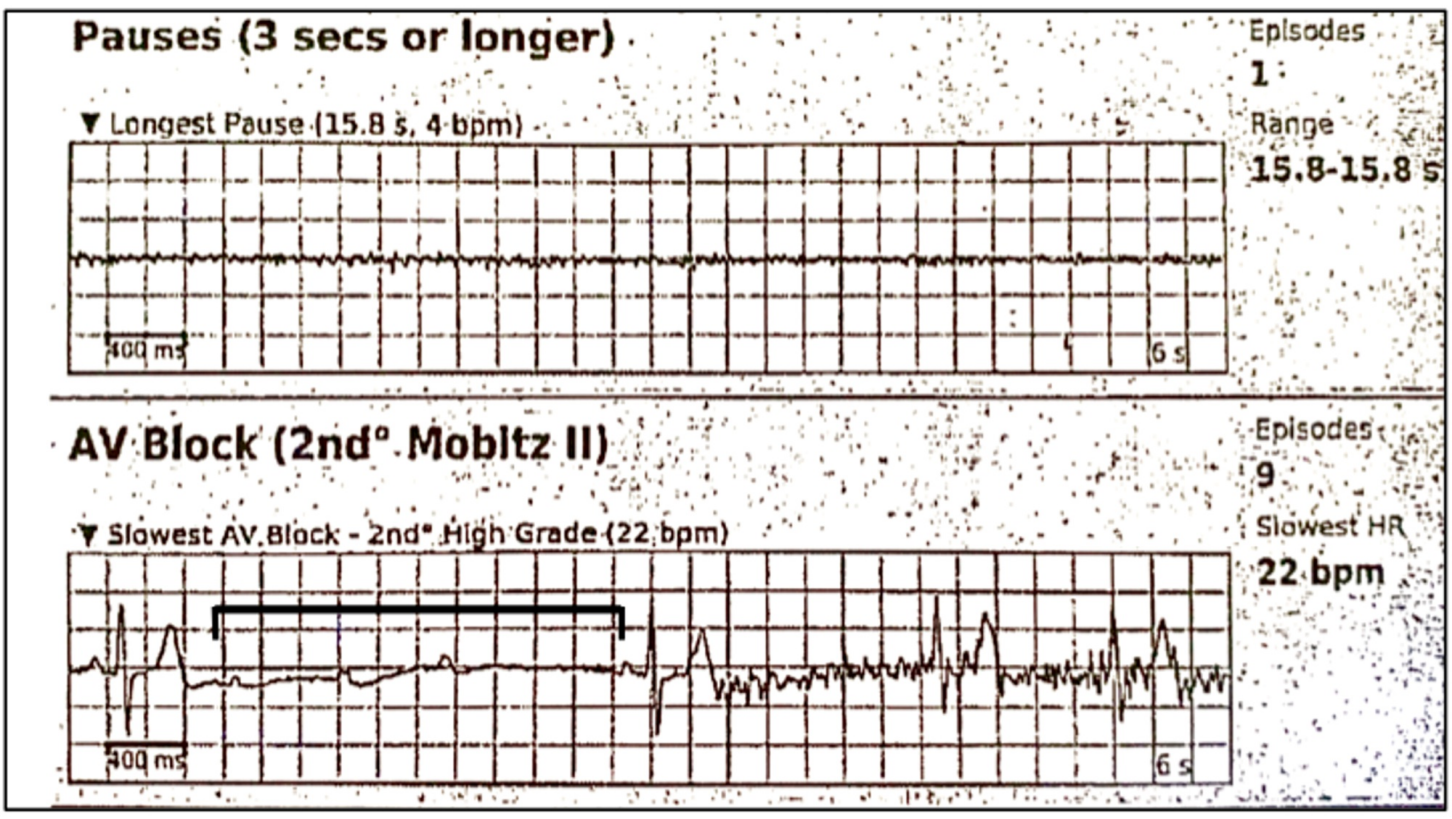
Holter monitor detection of high-grade AV block type II (Mobitz). The lowest heart rate recorded was 22 bpm AV: atrioventricular

The Holter monitor revealed multiple episodes of second-degree AV block Mobitz II, high-grade (Figure 2). The longest asystole event lasted 15.8 seconds and the shortest lasted 12.7 seconds. Second-degree AV block Mobitz I (Wenckebach), ventricular bigeminy, and trigeminy were also present. The predominant underlying rhythm was normal sinus, with a minimum heart rate of 22 beats per minute and a maximum of 154 beats per minute. The patient had 1.7% of PVCs (“occasional”). The longest ventricular bigeminy episode lasted 6.3 seconds whereas the longest trigeminy episode lasted 39.7 seconds.

On the day of the longest asystole event, the patient experienced a syncopal episode while urinating at home. The patient denied straining but admitted to chest pain and palpitations during the event. She reported a loss of consciousness and woke up on the floor. Her partner had witnessed the event and confirmed that she had been unconscious for a few seconds. She denied loss of bladder or bowel function, and she was not noted to have any tonic-clonic activity per her partner.

A 15.8-second pause, without a ventricular escape rhythm, suggested the presence of complete heart block. A dual-chamber pacemaker was recommended and a referral to an electrophysiologist (EP) was made.

The following day, the patient met the EP cardiologist who subsequently recommended a pacemaker implant due to a multitude of symptoms: syncope with mild trauma, daily recurrent presyncope events, presence of complete heart block, and high-grade type II AV block. She was admitted to the telemetry floor and was counseled about the potential risk to the fetus while undergoing sedation and fluoroscopy. Standard radiation precautions were taken; however, an additional shield was used for the gravid uterus. A St. Jude’s dual-chamber pacemaker (Abbott Laboratories, Abbott Park, IL) was implanted without complication.

Clinical Course

This patient’s dual-chamber pacemaker was set to pace at 60/100 beats per minute. At a routine follow up, the pacing was utilized at 11%, likely due to her high-grade heart block. The patient admitted to infrequent, yet mild episodes of shortness of breath, rare chest pain, and denied any further presyncope or syncopal episodes. Her symptoms were attributed to a growing fetus and gravid uterus compressing the inferior vena cava.

At 37 3/7 weeks, the patient presented to the labor and delivery unit with painful contractions without rupture of membranes. Due to non-reassuring fetal heart rate monitoring, the patient and her obstetrician elected to proceed with a cesarean section. Spinal anesthesia was given, and electrocautery precautions were taken with regard to her pacemaker use. A healthy baby girl weighing 3,700 grams with Apgar scores of 8 and 9 at one and five minutes, respectively, was born.

During labor, the patient received penicillin G due to her first-trimester RPR lab result. After delivery, the infant spent one night in the neonatal ICU (NICU) for hypoglycemic episodes. While in the NICU, it was noted that the baby had an RPR titer of 1:2, for which she received penicillin G. No congenital defects, including cardiac, were noted. The patient and her infant were discharged on postoperative day three with instructions for routine follow-up with her obstetrician, pediatrician, and an infectious disease physician for the treatment of her latent syphilis infection.

## Discussion

Background

**Figure 3:**
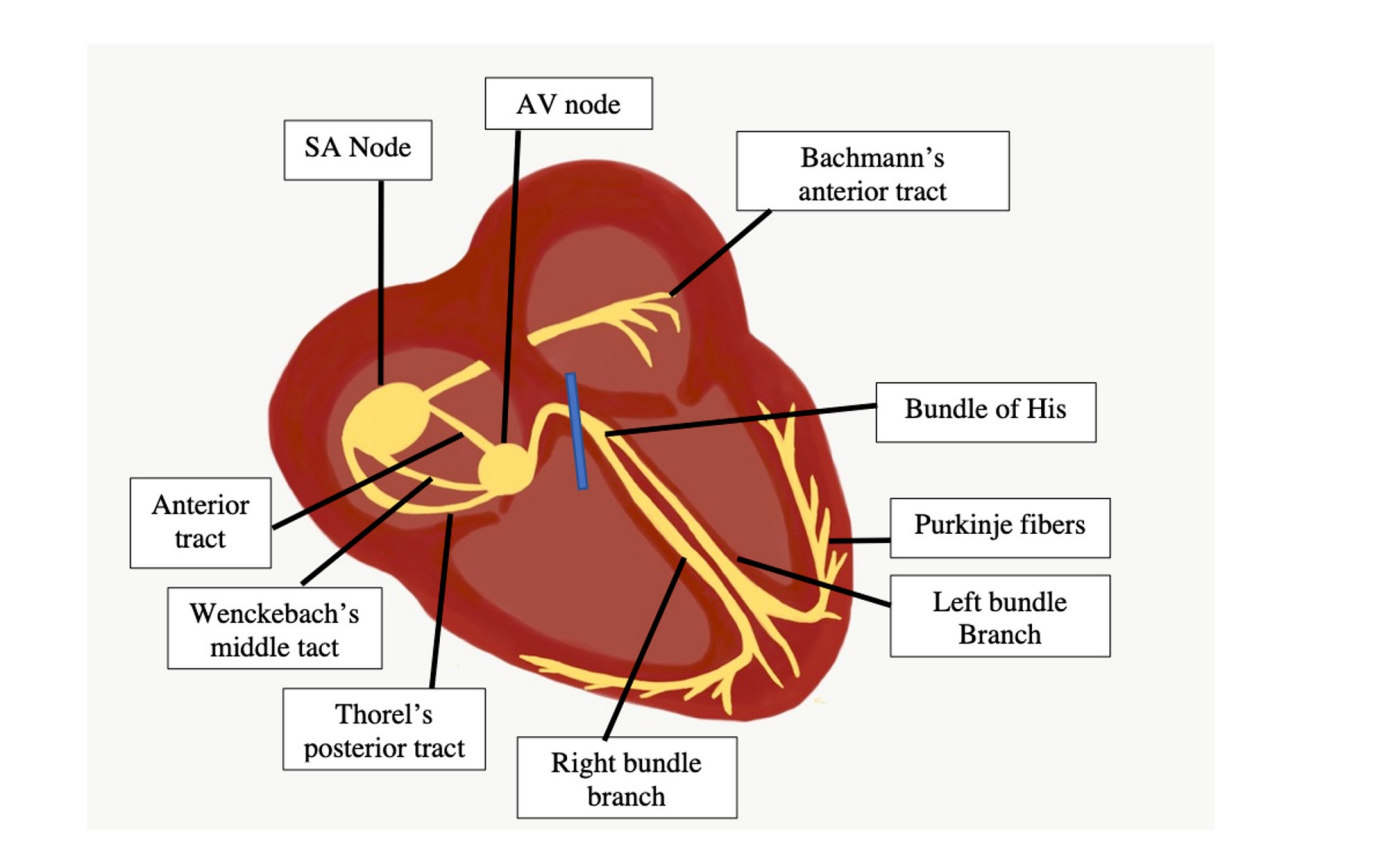
Electrical conduction of the heart Atrioventricular (AV) conduction provides the communication point between atria and ventricles, which eventually allows for myocardial ventricular contraction. AV block refers to a delay in the transmission of the electrical signal between the atria and ventricles. The location of the delay, which can include the AV node, bundle of His, and bundle branches, helps dictate the type of block and the outcomes of the delay. While first and second-degree type I Wenckebach tend to cause delay at the AV node, second-degree type II Mobitz and third-degree tend to be associated with delay below the AV node. The blue marker at the bundle of His along with the inferior Purkinje fibers denotes the possible locations of the AV block in our patient SA: sinoatrial

Patients with any degree of AV block typically complain of dizziness, decreased energy, palpitations, presyncope, or syncope. Those with more advanced disease might present with fatigue, angina, and congestive heart failure, reflecting inadequate cardiac output or tissue perfusion [2]. The most severe symptom is the Stokes-Adams attack, which is defined as syncopal attacks caused by polymorphic ventricular tachycardia [2]. The degree of an AV block can be distinguished based on ECG findings, anatomic site of the block (Figure 3), onset, severity, clinical presentation, underlying etiology, or associated conditions [2]. Of note, the generic term “high-grade AV block” is used to describe any form of AV block that suggests an increased risk for complete heart block (CHB) or symptomatic bradycardia. It typically includes type II second-degree block, 2:1 AV block, and CHB [2], and therefore should be avoided due to the various etiologies.

First-degree AV block is defined as a prolonged PR interval >200 milliseconds. It is typically thought to be a benign disease and may or may not be symptomatic. Symptoms may only be seen during times of exertion. A permanent pacemaker is typically not required [2].

Second-degree AV block can be classified as type I (Wenckebach) or type II (Hay). Second-degree type I (Wenckebach) is characterized by progressive prolonged PR intervals until a QRS complex is dropped. Type II is defined as a single non-conducted sinus P wave associated with fixed PR intervals before and after the blocked beat. The sinus rate is stable, as displayed on an ECG by constant PP intervals, and has at least two consecutive conducted P waves to determine the PR interval. Additionally, 70% of cases of type II second-degree AV block have a prolonged QRS complex and are localized within the His-Purkinje system and therefore permanent pacing is indicated [2].

The diagnosis of CHB is made with the demonstration of complete dissociation between atrial and ventricular activation [2]. High-grade is defined as 3:1, 4:1, or higher AV ratios. The block may be localized anywhere in the conduction system, but it is typically in the distal His bundle in 70-90% of cases. An escape rhythm in CHB may be generated by AV junction, His bundle, bundle branches, or distal conduction system; it rarely arises from the ventricular myocardium [2]. The site of origin on the escape rhythm in advanced AV block cases is essential as it determines the need for permanent pacing. Symptomatic AV block requires pacing regardless of the site, morphology, or rate of escape rhythm [2].

Acquired AV block may be secondary to many causes of myocardial scarring including but not limited to atherosclerosis, dilated cardiomyopathy, hypertension, infiltrative cardiomyopathies, inflammatory disorders, infectious diseases, post-surgical correction of cardiac valves, or coronary artery bypass grafting (CABG) [2]. Other sources include congenital complete heart block, maternal lupus, electrolyte imbalance, drug toxicity, Lyme disease, sarcoidosis, and amyloidosis [3]. Most cases are due to idiopathic causes; this does not affect management significantly in that most cases require a pacemaker regardless.

Pregnant women undergo several adaptive changes in the cardiovascular system throughout pregnancy. By 24 weeks' gestation, the same gestational age as our patient, circulating blood volume has usually increased by 50%, red cell mass by 20%, and cardiac output by 30-50%. The systemic vascular resistance has reached its nadir at a 30% decrease. The increase in cardiac output results from increases in both stroke volume and heart rate. Heart rate increases by 25% (equal to 10-20 beats per minute) compared to pre-pregnancy rates [3].

Differential diagnosis

For a pregnant patient at 24 weeks' gestation with palpitations and fatigue, the most common etiology would be normal pregnancy symptoms. As stated above, the second trimester of pregnancy at viability includes a significant increase in circulatory volume, which increases the stretching of the heart, potentially causing abnormal rhythms, such as PVCs seen in our patient.

The most common arrhythmia in women of childbearing age is paroxysmal supraventricular tachycardia [2]. Bradyarrhythmias in pregnancy are rare with a prevalence of only 1/20,000 and are usually caused by sinoatrial disease or congenital complete heart block [3]. Additionally, postural orthostatic tachycardia syndrome (POTS) and other AV blocks, such as second-degree type I with a relatively long Wenckebach, may be confused with second-degree type II block [2]. Essential investigation for suspected arrhythmias should include [3]:

· Resting 12-lead ECG

· ECG recorded during arrhythmias - Holter monitor or Zio patch

· Echocardiogram

· Laboratory analysis including complete blood count with differential, complete metabolic panel, thyroid function tests

Other etiologies include hypotension, seizures, and psychogenic causes, such as vasovagal syncope [4]. A thorough history, physical exam, and appropriate workup can delineate an etiology.

Analysis

Our patient was found to have significant conduction blockade and presented with signs and symptoms of inadequate perfusion. Though the etiology of her AV block remains unknown, we have two theories: one being an underlying congenital heart block manifesting de novo during pregnancy. In 30% of congenital heart block patients, first symptomatology occurs during pregnancy [5], likely due to the hyperdynamic circulation of pregnancy. Another theory is founded in her history of syphilis. Though it had been treated prior to her current pregnancy, her RPR titers were noted to be elevated during labor with resulting transmission to her child. 

Interestingly, our patient had a previous, full-term pregnancy a few years prior without complication. Prior to this routine prenatal visit, she denied having symptoms of dyspnea, orthopnea, angina, or difficulty breathing. Lastly, her echocardiogram with a normal ejection fraction and myocardial chamber size ruled out any hypertrophic or heart failure conditions. Vague, non-specific symptoms such as fatigue, dyspnea, and presyncope could still be attributed to a normal pregnancy due to volume expansion and a gravid uterus. However, in our case, a cardiac workup proved worthwhile as additional complaints of palpitations, presyncopal episodes, and eventually, one syncopal event suggested the possibility of something more serious.

Heart block in pregnancy is infrequently encountered, and as such, there are no established guidelines for the management of these patients. It requires a multidisciplinary approach involving the obstetrician, neonatologist, cardiologist, and anesthesiologist. Pregnant women with persistent bradycardia can be divided into two groups: asymptomatic and symptomatic (syncope, presyncope, dizziness, dyspnea). Those who are asymptomatic and are hemodynamically stable during pregnancy, labor, and postpartum may not require a permanent pacemaker. However, the EP cardiologist should be on standby in case the need for temporary pacing presents itself [5,6,7].

For symptomatic patients, atropine can be given on a short-term basis until a permanent pacemaker can be implanted. Due to the significant mortality associated with high-grade AV block, a permanent pacemaker should preferably be implanted. It is ideally placed pre-pregnancy, but it can be placed in the first and second trimesters as well. All emergent cases that present late in pregnancy or during labor should be counseled for a temporary pacer followed by a permanent pacer in the postpartum period. Thamen et al. described a small study of 26 pregnancies in which resolution of AV block occurred postpartum for some pregnancies, implying that pregnancy-related hemodynamic shifts may affect the conduction system [7,8]. Therefore, re-assessment of symptoms should be performed prior to permanent pacer implantation [7].

A review of the literature provides few guidelines or safety protocols for the implantation of a pacemaker during pregnancy. Due to the use of fluoroscopy and the associated radiation during the procedure, there is a risk of teratogenicity. Studies have shown that the risk of radiation-induced adverse events, such as fetal defect or miscarriage, increases proportionally as gestation continues up to 20 weeks. After that, an ionizing radiation dose of up to 100 mSv should not adversely affect fetal growth for the remainder of the pregnancy [9]. 

The harmful effects of radiation may be mitigated by the use of a protective lead shield covering the gravid uterus. Radiation exposure during pacemaker implantation, even with shielding, can range from 1.4-1.7 mSv [9]. It should be noted that the fluoroscopy system can be manipulated during the placement of a pacemaker, such that the radiation amount can be altered without affecting image quality significantly [10]. This would be beneficial in a pregnant patient. Alternative approaches to avoid the use of fluoroscopy and radiation include ECG-echocardiographic guidance or transesophageal echocardiography to guide the lead wire [5,8]. Regardless of the approach utilized, after implantation, an ECG and echocardiogram should be performed and the patient should be alerted to note any new onset of symptoms such as palpitations, shortness of breath, syncope, seizure, dizziness, or exercise intolerance [10].

Concerning labor and delivery, uterine contractions (i.e., Valsalva maneuver) displace blood into the central circulation, leading to increased maternal cardiac output. This could lead to reflex bradycardia, further worsening AV nodal blockade in heart disease patients [11]. However, overall, those with a pacemaker who underwent vaginal delivery tolerated labor stress well, and having a pacemaker is not necessarily an indication for a cesarean section [7].

If a cesarean section is employed whether for maternal or fetal indications, the use of spinal anesthesia during labor and/or cesarean section may result in a high sympathetic blockage resulting in bradycardia, which could worsen symptoms for women with heart block, especially those who are not paced. The use of incremental epidural top-ups with a concentrated solution or low-dose combined spinal-epidural anesthesia may avoid this complication [5]. Additionally, the use of high-voltage electrocautery (i.e., Bovie/monopolar) intraoperatively may result in pacemaker dysfunction whereas bipolar diathermy can be used safely in these patients [5].

Compared to women without heart disease, pregnant women with heart disease have a greater risk of fetal and neonatal complications, likely due to uteroplacental insufficiency from the reduction in maternal cardiac output. These complications include miscarriage, fetal or neonatal death, low birth weight, small for gestational age, intrauterine growth restriction (IUGR), and premature birth. Fetal surveillance is thus recommended with growth ultrasounds and non-stress testing [5,10,12]. Of note, there is no interference with the pacemaker from external fetal heart rate monitoring and ultrasonographic modalities. Lastly, the involvement of a maternal-fetal specialist, cardiologist, and EP cardiologist throughout the remainder of the pregnancy is advised [10].

Key points for clinicians 

$\cdot$ The obstetrician should have a clinical index of suspicion for underlying cardiac defects when “normal” pregnancy symptoms cannot explain the patient presentation; a prompt referral to a cardiologist should be initiated.

$\cdot$ The EP cardiologist should consider alternative approaches for implantation of a pacemaker in a pregnant patient, including a lead shield for the uterus, alteration of the fluoroscopy to minimize exposure to the fetus, and ECG-echocardiographic guidance or transesophageal echocardiography.

$\cdot$ The remaining pregnancy, labor, and delivery of a pregnant patient with a pacemaker is typically well-tolerated and the neonatologist should monitor the neonate for potential long-term uteroplacental insufficiency complications.

## Conclusions

Our previously healthy, 24-year-old G3P1011 patient presented with vague signs and symptoms of a normal pregnancy. Further consultation with a cardiologist was prompted when increasing fatigue and frequent palpitations continued to occur. Workup revealed high-grade second-degree AV block diagnosed via a 14-day Holter monitor. Immediate implantation of a dual-chamber pacemaker was performed under sedation and fluoroscopy. The remainder of her pregnancy and delivery were uneventful and resulted in a healthy baby girl being delivered via cesarean section for obstetric indications.
